# Development of rural tourism in China: The tragedy of anti-commons

**DOI:** 10.3389/fpubh.2022.939754

**Published:** 2022-10-13

**Authors:** Xinyi Wei, Qiuguang Hu

**Affiliations:** ^1^School of Business, Ningbo University, Ningbo, China; ^2^Dong Hai Strategic Research Institute, Ningbo University, Ningbo, China

**Keywords:** recreation industry, rural tourism, environmental development, anti-commons tragedy, governance path

## Abstract

The development of rural tourism presents the complexity of the industry, environment, governance, and coordination structure. In tourism development, due to the high dispersion and fragmentation of property rights of the resource objects themselves, as well as the involvement of many “atomic” property rights and other stakeholders, coupled with the lack of strong organization and leadership, the full development and utilization of rich tourism resources in rural areas is becoming problematic. It is difficult to realize the “rental value” of tourism resources, which leads to the tragedy of anti-commons. Taking the case of China, this paper explores the occurrence of anti-commons tragedy in the development of rural tourism, identifies the problems caused by the tragic governance of the anti-commons in the current environmental development, and further examines the governance path of the anti-commons tragedy in the tourism sector. The purpose of the study is to optimize the development environment of rural tourism and build a healthy space suitable for industry, residence, and tourism.

## Introduction

The development of rural tourism has become an important way to promote the development of the tourism industry, especially in rural areas with abundant ecological and cultural resources alongside beautiful and pleasant environments. However, due to the decentralization of rural resources and the fragmentation of property rights coupled with the serious issue of empty villages, rural tourism development often faces problems such as high cost of resource development and difficulties in low-income household coordination, resulting in a serious tragedy of anti-commons characterized by idle and wasted resources (1). The term tragedy of anti-commons was first put forward by American property rights legalist Michael Heller in 1998. It was the situation when due to the existence of many property owners with exclusive rights in the resources of the Commons, the pursuit of profit maximization by individuals prevents others from effectively using the resources of the Commons, resulting in the dilemma of waste and low utilization of resources (2). Currently, a few villages in China are fragmented as a result of excessive dispersion of property rights where the tragedy of anti-commons due to underutilization of public resources and insufficient supply of public welfare in rural tourism development has become increasingly prominent (3, 4). The “tragedy of anti-commons” studied in this paper is mainly defined as the “tragic” problems of the high cost of environmental development, waste, and destruction of rural resources caused by the contradiction between the institutional system and stakeholders in the construction of recreation and tourism in some Chinese villages. For example, the construction team of Suiyang Ming Village in Guizhou province of China staged a “contest” of interests between the construction team and the resident farmers that ended up with the time cost and construction cost becoming far higher than the objective value. Similarly, the Tangshan Village in Guizhou had planned to carry out tourism development, but developers withdrew their capital after farmers bid up the land transfer price (5). Therefore, it is necessary to analyze the drivers of anti-commons tragedy in rural tourism development and explore effective ways to address it.

## Reasons behind the tragedy of anti-commons

The tragedy of anti-commons in rural environment development is common. The main reason is the fragmentation of property rights which strengthens the degree of exclusivity resulting in the waste of resources that cannot be effectively used.

### Decentralization of resources

There exists a contradiction between the integrity of rural tourism development and the decentralization of tourism resources. The scale of the economy of enterprises engaged in tourism development requires that there is continuous and overall development, but the tourism resources are scattered, or even staggered with general resources with significant spatial division, which makes it difficult to carry out overall development as planned (6). Suiyang Ming village land property rights between public and private holdings were difficult. There were many real estate disputes and the village farmers and the construction team were engaged in a “protracted war.” The scattered land resources occupied by the construction team needed to be negotiated with the farmers according to the issue of land property rights. However, it proved to be difficult for scattered landowners to reach an agreement with the developers, which made the progress of rural tourism development very difficult.

### Fragmentation of power and responsibility

There is a contradiction between the integrity of rural tourism operation and management and the fragmentation of rural resource property rights. Tourist operators and management need unified standards, but because the control of rural resources is in the hands of a large number of farmers, it is very difficult to arrive at unified planning and management. The fragmented plots and scattered property rights are the main factors restricting the development and construction of rural tourism, and the resulting small-scale decentralized operation is inevitable (7). Longtou Village in Shanxi province mainly relied on farmers to operate small workshops, undertake responsibilities, and manage the operations. Due to the lack of unified operation and management, the small workshops gradually suffered after the initial prosperity, and further industrial development was stalled.

### Conflict and competition of interests

A contradiction exists between the overall income of rural tourism and the individual income of tourism stakeholders. In the development of rural tourism, due to the different resources, strengths, and interests, differences in income and competition for interests is inevitable (8). At times, the land transfer price is too high and exceeds the affordability of the requisitioned party, resulting in the land remaining idle and undeveloped. Further, unequal land distribution and interests are the fundamental causes of the differences between the two sides (9). Tangshan Village in Guizhou province had disputes and conflicts with farmers regarding the transfer of land for tourism development and finally had to stop production and shelved the high-quality rural resources.

### Individualization requirements of environmental development conditions

A contradiction also exists between the full regional requirements of rural tourism development and the personalized requirements of the stakeholders. Rural tourism is all about providing a specific kind of mood and atmosphere, which needs to be planned and developed from a comprehensive perspective. However, to promote environmental development, tourism development entities have to meet the individual requirements of stakeholders, which results in inconsistency in the overall styles of scenic spots and reduces the tourism experience. In Tangxi, Shangtian, and Zhangzhai villages in Wenzhou of Zhejiang province, due to low resettlement fees and insufficient compensation for housing foundations, individual farmers refused to combine their resources with tourism development and management, resulting in the shelving of development projects, which in turn affected the expected development schedule.

## Managing the tragedy of anti-commons tragedy

The contradictions between the whole and the individual, the dispute of resources, the competition of location, and competing interests are all important influencing factors. In addition, the development of the rural environment is also faced with many problems.

### Difficult to balance the income distribution

Farmers cannot obtain financial stability from land income alone and therefore are inclined to be cautious about issues relating to land property rights and transfer. In the development of rural tourism, the status of both parties in the transaction is often unequal, and enterprises are in the dominant position with relatively comprehensive market information, while the farmers are in a relatively weak position, insensitive to transaction information, low attention to market information, and weak negotiation skills. Enterprises and farmers consider their own interests, which are ultimately prone to opportunistic behavior, leading to the failure of transactions between both sides. The inequality of interest distribution in rural tourism development creates conflicts, resulting in problems such as construction plans being shelved and poor negotiations. Moreover, the conflict of interest arising from environmental development cannot obtain targeted and specialized legal assistance, which further deepens the conflict.

### Weak sense of integrity

Opportunistic behaviors such as “beating a bamboo stick” and “hitchhiking” in rural tourism development are the manifestation of both enterprises and farmers not establishing integrity and legal consciousness. The weak integrity awareness of farmers affects the relationship between enterprises and farmers and hinders the development progress of the rural environment. This is manifested as “nail households,” “principal households,” and “condition households” which hinder the development of rural tourism in various ways. Meanwhile, enterprises with insufficient integrity awareness and legal awareness violate the contract or display “edge ball” behavior. This behavior of obtaining individual income by damaging the interests of each other threatens the potential cooperative relationship between the two sides negotiating the process of rural tourism development. In the process of land transfers, opportunistic behaviors often occur among stakeholders due to weak legal awareness. For example, driving up the land price leads to excessive development costs for enterprises and violates the effective contract to seek individual interests.

### Imperfect rural governance mechanism

Development enterprises are generally approached through the village committee, collective economic organizations, and other representatives of the land circulation negotiations, by the village committee members or the person in charge of the organization who will integrate the land use of farmers, and finally, the two sides agree to sign an agreement. The government has the right to allocate social resources and gives the management right of enterprises with professional development potential, to ensure the smooth progress of development. However, enterprises may conduct “rent-seeking” to successfully win the bidding project and establish private contact with relevant personnel. In this process, there is a phenomenon that village members purchase land at a low price, and then transfer it to enterprises at a high price to obtain the intermediate price difference. Or the development enterprises and the village committee organization reach an agreement concealing the actual income of the land transfer, thereby violating the farmers' rights and interests.

### Hollowed-out rural economy

In the countryside, most of the farmers in the village are the elderly and children as the young men go out for work, resulting in a shortage of agricultural labor. Since farming and its related activities cannot achieve large-scale production, some cultivable land is abandoned, and housing also reflects a pattern of “outside expansion and inside empty.” The shortage of available labor in the village, the lack of human resources to drive development, coupled with the absence of a unified economic organization to manage the rural environment and the need for overall planning in the village, not only causes a lag in industrial development but seriously impacts the agricultural sector and economy. The hollowing out of the countryside also makes rural households indifferent to the development of the village environment, which further exacerbates the high cost of environmental development and the difficulty in organizing labor thereby seriously restricting the progress of rural environmental development.

### Constrained property rights system

The relevant legal guarantee of rural land property rights is vaguely defined. Except for the land use right and contracted management rights of the “four wastelands” (wasteland, waste mountains, sandy waste, and waste gullies), the land use right collectively owned over cultivated land and homestead shall not be subject to financial transactions such as a mortgage in principle. The transfer of rural homesteads must be carried out within the collective economic organization, thus limiting the transfer methods and channels of collective construction. In addition, according to the household contract responsibility system implemented in rural areas, a single family has a small amount of land and cannot form scale benefits. The imperfect legal guarantee system of related land management and possession leads to a variety of contract disputes (10), which seriously limits innovation and further hinders the involvement of land, property rights, and other issues in environmental development.

## Tragedy of anti-commons: Future pathways

Interest disputes, cooperation quality, village management, rural hollowing out, and ambiguous property rights are the main reasons for the anti-commons tragedy. Therefore, future pathways can be established by addressing these five challenges by adjusting interests in the relationship, improving cooperation consciousness, strengthening village organization management, promoting rural economic development, and clarifying the ownership relationship of property rights.

### Innovate the interest connection mechanism

Protecting the rights and interests of farmers is the key to addressing the tragedy of anti-commons. Therefore, it is necessary to improve the living standards of farmers, weaken the conflict of interest, give play to the third-party role of government supervision and management, establish an equal interest distribution mechanism, coordinate the conflict of interest among cooperation subjects, and improve the formulation of relevant laws. There is a need to innovate the distribution method to benefit all. For example, enterprises can use funds and stock to establish connections with farmers' land property rights, and housing property rights, and expand the distribution methods of interest. By establishing a third-party supervision balance system outside the transaction subject, the interest distribution mechanism can be objectively judged to guarantee an equal interest relationship. The third party could also play the role of coordination and supervision. Improving the legal guarantee in the transfer of farmers' land property rights can ensure that farmers enjoy relevant rights, and effectively avoid transaction conflicts between the two sides.

### Enhance the integrity and legal awareness of farmers

Due to the unequal cooperative relationship between farmers and enterprises, farmers who have a weak ability to grasp market information and are at a disadvantage will spontaneously set up protective barriers. To resist unequal status and impaired equity allocation, farmers may adopt a “pre-emptive” strategy to obtain some benefits. Therefore, the relevant negotiation, appeal, rights, and other information between farmers and enterprises should be open and transparent. Unless there are special circumstances, the terms and agreements must be strictly implemented, and a third party should cooperate and supervise. The village should regularly organize legal knowledge workshops on environmental development in the village committee or organization and encourage both enterprises and farmers to learn and know how to use the law to safeguard their rights and protect common interests. At the same time, stakeholders should establish legal awareness, pay attention to punitive measures for those “violating” the contracts, and safeguard the rights and interests of both sides by signing fair agreements.

### Optimize the rural governance mechanism

Government departments should take the lead, strengthen the supervision of rural tourism development, and improve the rural management system. In terms of institutional safeguards, the counterbalancing role of government supervision should be strengthened. Rules and regulations should be set up for rent-seeking of enterprises, and penalties should be factored into by law to prevent the occurrence of corruption. In terms of system guarantee, the phenomenon of multiple approvals should be eliminated in the development of rural tourism, and the cumbersome approval system should be standardized and simplified. Effective management and supervision of relevant procedures will ensure that transactions are open and transparent, while at the same time implementation of checks and balances would deter bad behavior. Establishing a rural development supervision organization as a third-party institution to supervise the cooperation subjects and stakeholders in the transaction will ensure the smooth progress of development and operation by overcoming various difficulties in tourism development.

### Strengthen the coordination ability of rural economic organizations

To give full play to the intermediary coordination ability of rural economic organizations, under the guidance of government agencies, tourism development coordination agencies should be established. These agencies should be mainly responsible for addressing issues such as the dispersion of property rights and blurred real estate boundaries in tourism development. As an intermediary bridge of cooperation and communication between the two sides, the tourism development coordination agency should coordinate the interest disputes and conflicts between enterprises and farmers. It should safeguard the rights and interests of both parties by supervising transaction behavior and promoting a harmonious cooperative relationship between them and strengthening the interest connection of both parties to prevent the occurrence of conflicts of interest. Continuously expanding the roles and responsibilities of rural economic organizations and strengthening their unified management and planning ability would be conducive to improving the utilization rate of rural agricultural development.

### Innovate the rural property rights system

It is necessary to establish a standardized land circulation mechanism, integrate market transaction information, and systemize the rules and systems of land circulation in rural tourism development. Equally, there is a need to adopt standardized operation and management under the market mechanism and cooperate with the invisible supervision of the government to ensure the openness and transparency of the land circulation market information as much as possible. The authorities should improve the rural land transaction system and formalize land circulation processes. To overcome the interest disputes arising from land transfer and the practical challenges that prevent resources from being effectively used, the contracts signed by enterprises and farmers must be first appraised by the supervision and management organization and then submitted to a third party for evaluation before being gradually promoted. Efforts must be made to learn and draw lessons from similar regulations and processes on property rights of idle houses abroad, increase idle tax according to actual idle housing, and optimize the land integration method by improving the laws and regulations on land circulation and collective economic property rights, to create a healthy environment for rural tourism development.

## Conclusion

The existing problems of the tragedy of anti-commons in some Chinese villages cannot be generalized and attributed according to the property rights of the anti-commons tragedies. It has been found that in the development and construction of rural tourism, many stakeholders are involved. Therefore, adjusting interests between these stakeholders, improving their cooperation consciousness, investing in farmers' integrity and legal awareness, strengthening village organization management, and clarifying the ownership relationship of property rights would be key to promoting the process of rural tourism development. An effective design scheme for the development of rural recreation tourism needs to establish a balance between stakeholders based on clarifying property rights and other systems.

## Data availability statement

The original contributions presented in the study are included in the article/Supplementary material, further inquiries can be directed to the corresponding author.

## Ethics statement

Written informed consent was obtained from the individual(s) for the publication of any potentially identifiable images or data included in this article.

## Author contributions

XW made intellectual contributions to the method and content of the manuscript. QH guided on topic selection, perspective, reviewed, and edited the manuscript. Both authors listed have made a substantial, direct, and intellectual contribution to the work and approved it for publication.

## Conflict of interest

The authors declare that the research was conducted in the absence of any commercial or financial relationships that could be construed as a potential conflict of interest.

## Publisher's note

All claims expressed in this article are solely those of the authors and do not necessarily represent those of their affiliated organizations, or those of the publisher, the editors and the reviewers. Any product that may be evaluated in this article, or claim that may be made by its manufacturer, is not guaranteed or endorsed by the publisher.
