# Supplementary material for: Development of rural tourism in China: The tragedy of anti-commons
Source: Front Public Health. 2022 Oct 13;10:939754. doi: 10.3389/fpubh.2022.939754 (PMC9610115; doi:10.3389/fpubh.2022.939754)
Supplement: Supplementary file 1 [file Data_Sheet_1.PDF]

frontiers

Supplementary Material

Reality case: The road construction tragedy in Suiyang Ming Village, Guizhou Province

As a pilot area of land reform, its rural land has not been adjusted from land division to household. Farmers will deal with agricultural land as "private property" in their specific life practice. Mingcun vigorously built village-level roads and group-level roads. Because of the problems involving land, all the roads could not be carried out under the condition of financial and labor conditions, because a farmer did not agree with the land being occupied. The best road scheme for the two groups is the direction of the dotted line in Figure 1.However, because every farmer who passes through uses the "private property right" of the land to obstruct it, they can only choose the route of the part of the real line, which is the tortuous and expensive worst scheme. The details are as follows:

The best road repair scheme for the two village groups (H and J) is the route according to the dotted line in Figure 1.(1) The main road at the village level goes to farmer A, and then bifurcates behind the house of Farmer A.(2) Group H passes directly behind the house of Farmer C along the dashed arrow.(3) Group J passes through farmland 1 to reach Group J. The sub-optimal scheme is to reach the J group through farmland 2, but because of the obstruction of the trading farmers, we can only choose the costly worst scheme.

First, in 2008, four farmers (A, B, C and D) jointly built the road from the village-level main road to farmer D's residence, with a length of about 190 meters. Therefore, the two groups H and J must first compensate the cost of the road for the construction of the road, through the "arduous" negotiations with nine farmers, and finally decide to compensate at the price of 2,000 yuan.

Secondly, group J plans to go through about 1 mu of farmland 1, and the road construction needs to occupy 0.5 mu, and the owner of farmland 1 works in other places. Field owner 1 put forward the requirement of "bundling", that is, if from farmland 1 passed, group J must "buy" all, and can not only occupy 0.5 mu, otherwise, refused to cooperate. Since Group J could not pay, a route passing through Farmland 2 was considered. Field owner 2 agreed to build the road plan, but because farmer A passed the land in front of the house, farmer A asked group H and J to compensate him 5,000 yuan. According to the market situation, the more reasonable land compensation cost is 2000 yuan, the two groups share the cost, but the farmers who do not cooperate are shown as "a penny can not be less".

Third, even if Group H can pass from Farmer A, it needs to pass through the house of Farmer C. But Farmer C requires Group H to follow the "D-B-B-C" route, otherwise the cultivated land behind Farmer C will not agree to "sell" to Group H. Farmer C proposes such a condition because he can get both road compensation and land transfer fees.

Fourth, when both Group H and Group J failed to agree with the farmers, the road repair scheme was forced to choose the solid line route in Figure 1.

Fifth, A section of about 15 m road from D to B was built by Farmer B. Group H compensated Farmer B RMB 3000, while a section of about 10 m road from B to C compensated Farmer C RMB 2000.

……

It took nearly a year to build the road because of all of these obstacles.In order to build the road can only meet all kinds of unreasonable requirements, and was forced to choose a high cost, poor design route scheme.According to the author's investigation, in Suiyang Ming Village, Guizhou Province, eight previous roads have experienced "anti-Commons tragedies".

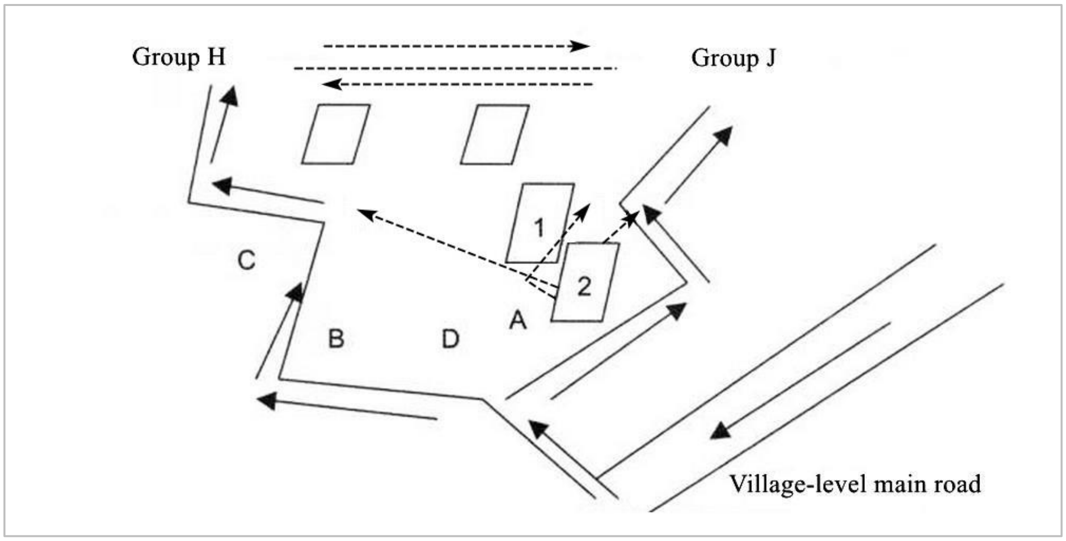

Figure.1 Road Construction Plan of Suiyang Ming Village
